# Evaluation of an *Aspergillus* IgG/IgM lateral flow assay for serodiagnosis of fungal asthma in Uganda

**DOI:** 10.1371/journal.pone.0252553

**Published:** 2021-05-28

**Authors:** Richard Kwizera, Felix Bongomin, Ronald Olum, William Worodria, Freddie Bwanga, David B. Meya, Bruce J. Kirenga, Robin Gore, David W. Denning, Stephen J. Fowler

**Affiliations:** 1 Infectious Diseases Institute, College of Health Sciences, Makerere University, Kampala, Uganda; 2 Makerere University Lung Institute, College of Health Sciences, Makerere University, Kampala, Uganda; 3 Department of Medical Microbiology, Faculty of Medicine, Gulu University, Gulu, Uganda; 4 Department of Medicine, School of Medicine, College of Health Sciences, Makerere University, Kampala, Uganda; 5 Division of Pulmonology, Mulago National Referral Hospital, Kampala, Uganda; 6 Department of Medical Microbiology, School of Biomedical Sciences, College of Health Sciences, Makerere University, Kampala, Uganda; 7 Cambridge University Hospitals NHS Foundation Trust, Cambridge, United Kingdom; 8 Division of Infection, Immunity and Respiratory Medicine, School of Biological Sciences, Faculty of Biology, Medicine and Health, The University of Manchester, Manchester, United Kingdom; 9 NIHR Biomedical Research Centre, Manchester University Hospitals NHS Foundation Trust, Manchester, United Kingdom; Clinic for Infectious and tropical diseases, Clinical centre of Serbia, SERBIA

## Abstract

**Background:**

Diagnosis of fungal allergies in asthma remains problematic in low-and middle-income countries due to non-availability of point-of-care testing. In this study, we aimed to evaluate the performance of an *Aspergillus* immunochromatographic technology (ICT) IgG/M lateral flow device (LFD) for the serological diagnosis of allergic bronchopulmonary aspergillosis (ABPA) and severe asthma with fungal sensitisation (SAFS) among Ugandan adult asthmatics.

**Methods:**

374 adult (aged ≥18years) asthmatics in the African Severe Asthma Program study, Ugandan site constituted the study population. ABPA and SAFS were diagnosed according to standard criteria. Asthmatics who did not meet the above criteria constituted a control group. The LFD tests were performed and read according to manufacturer’s instructions.

**Results:**

ABPA was found in 12/374 (3.2%) and SAFS in 60/374 (16%) participants. The sensitivity, specificity, positive predictive value (PPV) and negative predictive value (NPV) for the *Aspergillus* ICT for the diagnosis of ABPA were 0.0%, 96.4%, 0.0% and 96.7% respectively, and for SAFS 6.7%, 97.1%, 30.8% and 84.5% respectively. False positive and negative rates were 3.5% and 3.2% for ABPA and 2.4% and 14.9% for SAFS, respectively. Patients with a positive LFD significantly had higher median *Aspergillus fumigatus*-specific IgE levels compared to those with negative LFD (median: 0.06 kUA/l VS 0.03 kUA/L, P = 0.011).

**Conclusion:**

The *Aspergillus* ICT IgG/M LFD had a poor diagnostic performance for the diagnosis of both ABPA and SAFS. Its greatest value may be in distinguishing chronic and allergic aspergillosis in Africa.

## Introduction

Airway exposures to fungal elements among patients with asthma elicits immune responses and bronchopulmonary inflammation, typically presenting as severe asthma with fungal sensitization (SAFS) or allergic bronchopulmonary aspergillosis (ABPA) [1]. *Aspergillus fumigatus* is the usual culprit in fungal sensitization complicating 15–50% of cases of bronchial asthma, and associated with poor disease control [2]. On a global scale, ABPA alone is diagnosed in 1 to 4% of adult asthma patients [3–7]. Asthma prevalence is estimated at 11% in Uganda [8]. Fungal asthma is a significant problem among Ugandans with asthma [9]. Our recent work has shown that approximately over 4 million adult asthmatics in Africa have fungal sensitization, with an estimated 437,000 presenting with ABPA and 577,000 SAFS [10].

Diagnosis of *Aspergillus* sensitization requires demonstration of evidence of allergic sensitisation to *Aspergillus* either by skin prick testing (SPT) or *Aspergillus*-specific IgE immunoassays [5, 11, 12]. Other tests such as *Aspergillus*-specific IgG, total serum IgE and eosinophil count can be added to get a definite diagnosis of ABPA as proposed by *Agarwal* and colleagues [13]. High resolution chest CT scan may be added to distinguish between “serological ABPA” and “ABPA with bronchiectasis” [14]. SAFS is diagnosed in a patient with severe asthma with evidence of fungal sensitization after ABPA has been excluded [15]. However, most of these approaches are hardly performed routinely in the resource-limited settings since they are costly and need referral laboratory facilities.

Recently, *Aspergillus* lateral flow devices (LFD) have been developed as new point of care diagnostic tests for the qualitative detection of *Aspergillus* antigens or antibodies in human serum and bronchoalveolar lavage (BAL) fluid [16, 17]. They were recently added to the World Health Organization (WHO) essential diagnostics list (https://www.gaffi.org/3rd-essential-diagnostics-list-launch/). The *Aspergillus* antigen test is recommended for the diagnosis of invasive aspergillosis (IA) [16, 18] while the *Aspergillus* antibody test is recommended for the diagnosis of chronic pulmonary aspergillosis (CPA) [19]. However, it is not yet approved for diagnosis of ABPA or SAFS.

A novel *Aspergillus* immunochromatography technology (ICT) IgG-IgM (LDBio Diagnostics, Lyon, France) test has been developed, and allows the simultaneous detection of both IgG and IgM class anti-*Aspergillus* antibodies in blood. It is based on the principle of the sandwich. It has been tested in France and the United Kingdom and might in fact be more useful in resource-limited setting because of its simplicity and cost [20, 21]. It has a sensitivity and specificity of 91.6% and 98.0% respectively for the serological diagnosis of chronic pulmonary aspergillosis (CPA) [20]. A recent study showed that the LDBio Aspergillus ICT has an excellent diagnostic performance for the serological diagnosis of ABPA [22]; however, the utility of this kit for the diagnosis of SAFS and ABPA in Africa has not been studied before. We therefore aimed to evaluate the diagnostic performance of the LD Bio *Aspergillus* ICT IgG-IgM lateral flow assay for the serological diagnosis of ABPA and SAFS among Ugandan adult asthmatics.

## Materials and methods

### Study design and population

This was a cross-sectional study evaluating the diagnostic performance of the LDBio *Aspergillus* ICT IgG-IgM lateral flow assay for the serological diagnosis of ABPA and SAFS among Ugandan adult asthmatics. This study was nested within the African Severe Asthma Program (ASAP) clinical study (*ClinicalTrials*.*gov Identifier*: *NCT03065920*) [23–25] at the Makerere University Lung Institute. ASAP is a clinical study with the primary objective being to identify and characterize severe asthma in Uganda, Kenya, and Ethiopia.

All participants in the current study were adults (≥18years) enrolled in ASAP at the Ugandan site having a “current/previous doctor diagnosis of asthma or clinical/treated asthma or wheezing/whistling breath in the last 12 months”. Patients with an alternative lung disease likely to confound assessment of asthma, those unable to perform study tests and procedures and pregnant women were excluded. Each patient was followed up for 12 months.

### Case definition for ABPA and SAFS

For this study, we used the case definition for SAFS and ABPA as proposed by ABPA complicating asthma International Society for Human & Animal Mycology (ISHAM) working group [13]. **Table 1** summarises this case definition (**Table 1**). Any patients who did not fit this case definition were taken as controls.

**Table 1:** Case definition for SAFS and ABPA.

*Aspergillus* sensitisation	Positive AF SPT or AF IgE
**ABPA**	Positive TIgE >1000 IU/mL and Positive AF SPT or Asp IgE and AEC>500 cells/μL
**SAFS**	Severe asthma and Fungal sensitisation (Positive Asp SPT or Asp IgE) and Positive TIgE <1000
**ABPM**	Severe asthma and Negative Asp IgE and Asp SPT and Positive TIgE <1000 Positive MM SPT

Data presented are Case definitions for fungal asthma. ABPA = Allergic bronchopulmonary aspergillosis, SAFS = Severe asthma with fungal sensitization, ABPM = Allergic bronchopulmonary mycosis, AF = Aspergilllus fumigatus, SPT = skin prick test, Asp = Aspergillus, IgG = Immunoglobulin G, IgE = Immunoglobulin E, TIgE = total Immunoglobulin E, AEC = absolute eosinophil count, MM = Mold mix.

### Study assays and cut-offs

Samples used for this study were stored baseline serum samples from the ASAP study population at the Ugandan site (stored at -80°C). Samples testing was performed via ImmunoCAP® (ThermoFisher, previously Phadia) for levels of *Aspergillus fumigatus* -specific IgG (Asp IgG), *Aspergillus fumigatus*-specific IgE (Asp IgE) and total serum IgE (TIgE) antibodies. We used cut-offs of 40 mg/l for the Asp IgG and 0.35 kUA/l for Asp IgE as recommended by manufacturer. We used a cut-off of 113.9 kU/L for total IgE as recommended by manufacturer and a cut-off of 1000 kU/L as a screen for ABPA [26]. A cut-off of 500 cells/ul for eosinophil was used for ABPA [13].

Skin prick testing (SPT) (Immunospec (Pty) Ltd, Johannesburg, Gauteng, South Africa) was performed at baseline for all patients, and the tests were performed and interpreted according to international guidelines [27]. We used a panel of 12 allergens that included *A*. *fumigatus*. Normal saline served as a negative control while histamine was the positive control, with a mean wheal diameter of at least 3 mm being positive, read after 15 minutes of allergen application.

### Procedure for *Aspergillus* ICT IgG-IgM test

The *Aspergillus* ICT IgG-IgM is a rapid lateral flow test and was performed according to manufacturer’s instructions. In brief, 15μL of patient’s sample were added to the LFD followed by four drops of sample diluent. This was left at room temperature and read after 20 minutes. Two trained laboratory technologists visually read the test results.

### Data analysis

Data were analysed using STATA version 14 (STATA, College Station, Texas). Descriptive statistics were used to summarize baseline characteristics including lung function of participants; categorical variables as frequencies (percentages) and numerical variables as median (interquartile range). Our primary data analysis aimed at establishing the diagnostic performance of the LD Bio Aspergillus ICT IgG-IgM lateral flow assay for the diagnosis of ABPA and SAFS among Ugandan adult asthmatics at a 95% confidence interval. We compared the Aspergillus lateral flow assay to confirmed ABPA and SAFS so as to calculate the sensitivity, specificity, positive predictive value (PPV), negative predictive value (NPV) and level of agreement, and receiver-operator characteristic (ROC) area under the curve (AUC) at a 95%CI. Mann-Whitney U test was performed to compare the levels of Asp IgG, Asp IgE and TIgE in patients with a positive and negative Asp LFD results.

### Ethical statement

Participants provided written informed consent to participate in the ASAP study (MHREC 875). Ethics approval for this sub-study was received from the school of biomedical sciences research and ethics committee (SBS 598), the Uganda National Council for Science and Technology (HS 2532) and the Uganda National Drug Authority (9464).

## Results

### Study population characteristics

Between May 2017 and June 2018, we enrolled 374 asthma patients in this study. Of 374 asthma patients included in the study, 286 (76.5%) were female and the median age for all participants was 34 years (IQR 25–45; n = 369) (**Table 2 and S1 Table**). Of the 374 participants, 143 (38.2%) had a positive SPT against *A*. *fumigatus*, 16 (4.3%) had elevated *A*. *fumigatus*-specific IgG levels, 27 (7.2%) had elevated *A*. *fumigatus*-specific IgE levels, 12 (3.2%) had proven ABPA while 60 (16.0%) had proven SAFS. Of the 302/374 controls without ABPA or SAFS, 85 had asthma with *Aspergillus* sensitization only, 11 had asthma with allergic bronchopulmonary mycosis (ABPM), while 206 had asthma without any form of fungal sensitization.

**Table 2:** Selected baseline characteristics of the study population.

	Overall (N = 374)		Intermittent & mild asthma (N = 88)		Moderate persistent & severe persistent asthma (N = 286)		
Characteristic	N	n (%) or median (IQR)	N	n (%) or median (IQR)	N	n (%) or median (IQR)	P-value
**Current age in years**	369	34 (25–45)	88	29 (23–41)	281	35 (26–46)	0.013
**Male Gender**	374	88 (23.5)	88	20 (22.7)	286	68 (23.8)	0.839
**Overall BMI**	374	24.8 (21.6–29.5)	88	23.7 (20.9–28.9)	286	25.7 (21.6–29.7)	0.104
**Mean ACQ**	372						
**Well controlled**		94 (25.3)	88	46 (52.3)	284	48 (16.8)	0.000
**Not well controlled**		278 (74.7)		42 (47.7)		236 (82.5)	
**Pre-measurements**			88		286		
**FVC**	374	2.9 (2.2–3.4)		3.3 (2.9–3.6)		2.7 (2.1–3.2)	0.000
**FVC%**	374	100 (83–112)		110 (103–120)		95.5 (78–109)	0.000
**FEV1**	374	2.1 (1.6–2.7)		2.6 (2.3–3.0)		1.97 (1.4–2.5)	0.000
**FEV1%**	374	88 (65–105)		103 (91.5–115.5)		79 (60–100)	0.000
**FEV1/FVC ratio**	374	0.76 (0.66–0.84)		0.82 (0.74–0.88)		0.73 (0.63–0.83)	0.000
**Post-measurement**			9		117		
**FVC**	126	2.6 (2.2–3.4)		3.1 (2.8–3.6)		2.62 (2.2–3.4)	0.050
**FVC %**	126	99.5 (80–112)		117 (111–120)		97 (80–111)	0.002
**FEV1**	126	1.8 (1.3–2.3)		2.3 (2.1–2.5)		1.73 (1.29–2.2)	0.010
**FEV1%**	126	73.5 (56–89)		98 (92–103)		71 (56–87)	0.000
**FEV1/FVC ratio**	126	0.65 (0.56–0.71)		0.74 (0.7–0.76)		0.64 (0.56–0.71)	0.006
**Eosinophil count**	374	210 (110–390)		200 (110–410)		215 (100–390)	0.984
**Patient uses ICS**	370	34 (9.2)	87	3 (3.4)	283	31 (11)	0.034
**Patient uses inhaler medication**	370	108 (29.2)	87	13 (14.9)	283	95 (33.6)	0.001

Data presented are percentages (%), medians and interquartile ranges (IQR). BMI = body mass index, FVC = Forced vital capacity, FEV = Forced Expiratory Volume, ACQ = Asthma Control Questionnaire, ICS = Inhaled corticosteroids.

### Comparison of *Aspergillus* lateral flow assay against ImmunoCap *Aspergillus* IgG, IgE and total IgE

**Fig 1:**
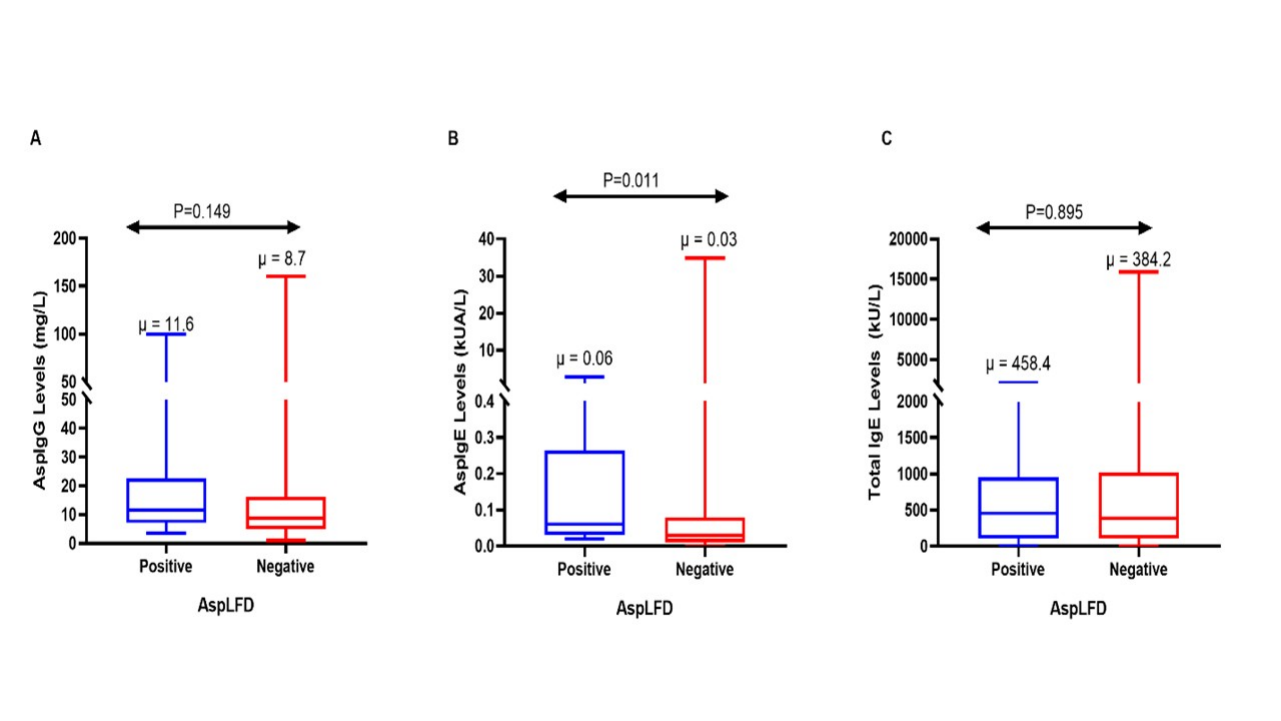
Distribution of levels of Asp IgG (A), Asp IgE (B) and Total IgE (C) in patients with a positive or negative LFD. μ- median.

Patients with a positive LFD significantly had higher median Asp IgE levels compared to those with negative LFD (median: 0.06 kUA/l VS 0.03 kUA/L, P = 0.011). The differences in Asp IgG and total IgE were not statistically significant (**Fig 1**).

### Performance of the *Aspergillus* lateral flow assay against ABPA

Using confirmed ABPA as the reference standard, the LDBio *Aspergillus* ICT IgG-IgM ICT had a sensitivity of 0.0% (0/12), specificity of 96.4% (349/362), PPV of 0.0% (0/13) and a NPV of 96.7% (349/361). We registered 13 (3.5%) false positives and 12 (3.2%) false negatives. There was a 93.3% agreement between the two tests (ABPA vs LFD). However, Cohen’s Kappa analysis [28] showed no level of agreement between the two tests (κ = -0.035, p = 0.748). The area under the ROC curve for sensitivity and specificity showed poor performance (C-statistic = 0.48, 95% CI: 0.47 to 0.49) for the LDBio *Aspergillus* ICT IgG-IgM lateral flow assay when compared to confirmed ABPA (**Table 3**). None of the patients with ABPA and 16 of the non-ABPA patients had an elevated *Aspergillus*-specific IgG levels. Similarly, 24 of the non-ABPA patients had an elevated *Aspergillus*-specific IgE levels.

**Table 3:** Summary of diagnostic performance of LD Bio *Aspergillus* ICT IgG-IgM lateral flow assay for the diagnosis of ABPA and SAFS.

Reference standard	Confirmed ABPA	Confirmed SAFS
**n/N**	12/374	60/374
**True positives**	0/12 (0.0%)	4/60 (6.7%)
**False positives**	13 (3.5%)	9 (2.4%)
**False negatives**	12 (3.2%)	56 (14.9%)
**Sensitivity 95%CI**	0.0% (0/12) (0.0% to 26.5%)	6.7%% (4/60) (1.9% to 16.2%)
**Specificity 95%CI**	96.4% (349/362) (93.9% to 98.1%)	97.1% (305/314) (94.6% to 98.7%)
**PPV 95%CI**	0.0% (0/13) (0.0% to 24.7%)	30.8% (4/13) (12.4% to 58.3%)
**NPV 95%CI**	96.7% (349/361) (96.6% to 96.7%)	84.5% (305/361) (83.5% to 85.4%)
**% Agreement 95%CI**	93.3% (90.3% to 95.6%)	82.6% (78.4% to 86.3%)
**Kappa statistic P-value**	-0.035 (P<0.748)	0.056 (P<0.070)
**C-Statistic 95%CI**	0.48 (0.47 to 0.49)	0.52 (0.49 to 0.55)
**Overall Diagnostic performance**	Poor	Poor

Data presented are the numbers, percentages, numerator/denominator, and 95% confidence interval. N = number of observations, ABPA = Allergic bronchopulmonary aspergillosis, SAFS = Severe Asthma with fungal sensitisation, SPT = skin prick test, PPV = Positive predictive value, NPV = negative predictive value, C- statistic = receiver-operator characteristic (ROC) area under the curve (AUC) for sensitivity and specificity. % agreement (Accuracy) = overall probability that a patient is correctly classified.

### Performance of the *Aspergillus* lateral flow assay against SAFS

Using confirmed SAFS as the reference standard, the LD Bio Aspergillus ICT IgG-IgM lateral flow assay had a sensitivity of 6.7%% (4/60), specificity of 97.1% (305/314), PPV of 30.8% (4/13) and a NPV of 84.5% (305/361). Nine (2.4%) false positive and 56 (14.9%) false negatives tests were observed. There was an 82.6% agreement between the two tests. However, Cohen’s Kappa [28] analysis showed no level of agreement between the two tests (κ = 0.056, p = 0.07). The area under the ROC curve for sensitivity and specificity showed poor performance (C-statistic = 0.52, 95% CI: 0.49 to 0.55) (**Table 3**). Two of the patients with SAFS and 14 of the non-SAFS patients had an elevated *Aspergillus*-specific IgG levels. Similarly, 22 of the non-SAFS patients had an elevated *Aspergillus*-specific IgE levels.

## Discussion

Determination of serological evidence of *Aspergillus* sensitization through an ELISA-based quantitative assay of total IgE and *Aspergillus*-specific IgE is an important component of the diagnostic criteria for both ABPA and SAFS [29]. However, these tests are often expensive, not available in most low- and middle-income countries (LMICs), labor intensive, and do require uninterrupted supply of electricity in an established infrastructural setting such as a reference laboratory [30]. These attributes limit their use in LMICs settings where they are most needed [31]. Therefore, there is a need to leverage existing point of care platforms such as the LDBio *Aspergillus* ICT to allow cheap and rapid serological screening for ABPA and other allergic pulmonary fungal diseases.

Here, we report our findings from the evaluation of a novel *Aspergillus*-specific IgG/IgM LFD for the serological diagnosis of ABPA and SAFS compared with a standard diagnostic criterion. Our study findings suggest that the LDBio *Aspergillus*-specific IgG/IgM has a poor sensitivity and should not be used alone for the diagnosis or screening for either ABPA or SAFS. None of the participants with ABPA and less than 7% of participants with SAFS had a positive LFD test. Yet, we previously demonstrated a 32% prevalence of *Aspergillus* sensitisation in the same Ugandan population [32]. However, in both patients with ABPA and SAFS, the LFD had excellent specificity (>96%) and very good NPV (>84%). This suggests that a negative LFD may be used as a rule out test for fungal allergies in patients with asthma. However, more studies are needed to evaluate its utility in this population. Interestingly, a recent evaluation of the same assay in Caucasian patients with ABPA showed a much higher sensitivity of 90.6%, and a specificity of 87.2% [22].

The performance of LDBio *Aspergillus*-specific IgG/IgM LFD has been studied for the diagnosis of IPA, ABPA and chronic pulmonary aspergillosis (CPA). In a multicenter European study among a diverse group of patients with pulmonary aspergillosis, the reported sensitivity and specificity was 88.9% and 96.3%, respectively [21]. Similar, among a cohort of 154 patients with CPA at the National Aspergillosis Centre in Manchester, UK, the reported sensitivity and specificity of the kit was 91.6% and 98.0%, respectively [33]. In Indonesian patients with proven CPA, LDBio LFD had a sensitivity of 80% and a specificity of 70% [34]. From these studies, LDBio kit has been demonstrated to meet the ASSURED (“affordable, sensitive, specific, user-friendly, rapid and robust, equipment-free, and deliverable to end users”) criteria outlined by the WHO [35].

Our study is not without limitations. The sample size could have been suboptimal. We did not include radiology which would help to rule out any cases of CPA complicating fungal asthma in this population. We used a single diagnostic criterion, yet the prevalence of ABPA varies with the diagnostic criteria or case definition used. A recent study revealed that new diagnostic criteria, principally a lower IgE level, compared with existing criteria, showed better sensitivity and specificity for diagnosing ABPA [36]. We likely over diagnosed some patients as ABPA since we only had available some of the key data required as criteria. Similarly, the high prevalence of SAFS could be related to the very high prevalence of severe asthma (76.5%) in this cohort, and thus may not truly reflect the prevalence of the disease among the general population of individuals with asthma in Uganda. However, the strength of our study in the use of data from patients with proven ABPA/SAFS using standard diagnostic criteria. Also, patients and controls were derived from a homogenous population–that is, we used diseased controls consisting of patients with asthma without ABPA or SAFS thus accurately representing the population and setting in which the test is most likely to be used.

## Conclusion

In conclusion, the LDBio Aspergillus ICT IgG-IgM lateral flow assay had a poor diagnostic performance for the diagnosis of both ABPA and SAFS in Uganda. However, with its high specificity, it could be used to rule out these disorders. It may be particularly useful in distinguishing chronic and allergic aspergillosis in Africa.

## Supporting information

S1 Table Extended baseline characteristics of the study population. (PDF)

S1 Dataset (XLSX)
