# Supplementary material for: Evaluation of an Aspergillus IgG/IgM lateral flow assay for serodiagnosis of fungal asthma in Uganda
Source: PLoS One. 2021 May 28;16(5):e0252553. doi: 10.1371/journal.pone.0252553 (PMC8162618; doi:10.1371/journal.pone.0252553)
Supplement: S1 Table — (PDF) [file pone.0252553.s001.pdf]

S1 Table. Extended baseline characteristics of the study population

	Overall (N=374)		Intermittent & mild asthma (N=88)		moderate persistent & severe persistent asthma (N=286)		
**Characteristic**	**N**	**n (%) or median (IQR)**	**N**	**n (%) or median (IQR)**	**N**	**n (%) or median (IQR)**	**P-value**
**Current age in years, median**	369	34 (25 - 45)	88	29 (23 - 41)	281	35 (26 - 46)	0.013
**Age when first diagnosed with asthma**	372	25 (15 - 36)	87	23 (15 - 33)	285	25 (15 - 36)	0.650
**Categories of current age in years**	367		87		280		
**18 - 24**		86 (23.4)		24 (27.6)		62 (22.1)	0.234
**25 - 34**		102 (27.8)		31 (35.6)		71 (25.4)	
**35 - 44**		82 (22.3)		15 (17.2)		67 (23.9)	
**45 - 54**		49 (13.4)		8 (9.2)		41 (14.6)	
**55 - 64**		37 (10.1)		7 (8)		30 (10.7)	
**65+**		11 (3)		2 (2.3)		9 (3.2)	
**Male Gender**	374	88 (23.5)	88	20 (22.7)	286	68 (23.8)	0.839
**Overall BMI**	374	24.8 (21.6 - 29.5)	88	23.7 (20.9 - 28.9)	286	25.7 (21.6 - 29.7)	0.104
**Underweight**		22 (5.9)		5 (5.7)		17 (5.9)	
**Normal**		168 (44.9)		50 (56.8)		118 (41.3)	0.040
**Overweight**		103 (27.5)		15 (17)		88 (30.8)	
**Obese**		81 (21.7)		18 (20.5)		63 (22)	
**Marital Status**	374		88		286		
**Single**		131 (35)		35 (39.8)		96 (33.6)	0.031
**Married**		188 (50.3)		44 (50)		144 (50.3)	
**Separated**		27 (7.2)		8 (9.1)		19 (6.6)	
**Widowed**		28 (7.5)		1 (1.1)		27 (9.4)	
**Highest level of education**	374		88		286		
**None**		14 (3.7)		2 (2.3)		12 (4.2)	0.001
**Primary**		118 (31.6)		16 (18.2)		102 (35.7)	
**Secondary**		133 (35.6)		46 (52.3)		87 (30.4)	
**Tertiary**		109 (29.1)		24 (27.3)		85 (29.7)	
**Occupational risk**	373		88		285		

**Low**		200 (53.6)		45 (51.1)		155 (54.4)	0.347
**Moderate**		123 (33.0)		34 (38.6)		89 (31.2)	
**High**		50 (13.4)		9 (10.2)		41 (14.4)	
**Cough**	374	170 (45.5)	88	31 (35.2)	286	139 (48.6)	0.028
**Duration in days**	170	7 (5 - 30)	31	7 (7 - 30)	139	7 (5 - 30)	0.716
**Wheezing**	374	152 (40.6)	88	24 (27.3)	286	128 (44.8)	0.004
**Duration in days**	152	7 (5 - 25.5)	24	10.5 (7 - 22)	128	7 (4 - 25.5)	0.267
**Chest Pain**	374	119 (31.8)	88	18 (20.5)	286	101 (35.3)	0.009
**Duration in days**	118	7 (7 - 14)	18	10.5 (7 - 16)	100	7 (3 - 14)	0.157
**Shortness of breath**	374	105 (28.1)	88	14 (15.9)	286	91 (31.8)	0.004
**Duration in days**	105	7 (3 - 14)	14	14 (7 - 30)	91	7 (3 - 14)	0.081
**Blocked Nose**	374	32 (8.6)	88	6 (6.8)	286	26 (9.1)	0.505
**Duration in days**	32	8.5 (5 - 30)	6	10.5 (5 - 30)	26	8.5 (5 - 30)	0.942
**Running nose**	374	22 (5.9)	88	8 (9.1)	286	14 (4.9)	0.144
**Duration in days**	22	12 (7 - 30)	8	7 (7 - 30)	14	14 (7 - 30)	0.422
**Sputum production**	374	22 (5.9)	88	3 (3.4)	286	19 (6.6)	0.312
**Duration in days**	21	7 (41760)	3	7 (11140)	18	7 (41760)	0.627
**Sore throat**	374	4 (1.1)	88	0 (0)	286	4 (1.4)	0.577
**Duration in days**	4	5 (2.5 - 18.5)	0		4	5 (2.5 - 18.5)	NA
**Level of asthma control**	372						
**Well controlled**		94 (25.3)	88	46 (52.3)	284	48 (16.8)	0.000
**Not well controlled**		278 (74.7)		42 (47.7)		236 (82.5)	
**Currently comorbidities**	374		88		286		
**Rhino sinusitis**		115 (30.7)		33 (37.5)		82 (28.7)	0.117
**Gastroesophageal reflux disease**		90 (24.1)		15 (17)		75 (26.2)	0.078
**Hypertension**		42 (11.2)		7 (8)		35 (12.2)	0.266
**HIV**		28 (7.5)		3 (3.4)		25 (8.7)	0.109
**Eczema/dermatitis**		23 (6.1)		3 (3.4)		20 (7)	0.311
**Obstructive sleep apnea**		10 (2.7)		1 (1.1)		9 (3.1)	0.463
**Depression or other psychiatry illness**		3 (0.8)		0 (0)		3 (1)	1.000
**Heart failure**		1 (0.3)		0 (0)		1 (0.3)	1.000
**Nasal polyps**		0 (0)		0 (0)		0 (0)	NA
**COPD**		0 (0)		0 (0)		0 (0)	NA
**None of the above**		171 (45.7)		42 (47.7)		147 (51.4)	0.385

**Pre-measurements**			88		286		
**FVC**	374	2.9 (2.2 - 3.4)		3.3 (2.9 - 3.6)		2.7 (2.1 - 3.2)	0.000
**FVC%**	374	100 (83 - 112)		110 (103 - 120)		95.5 (78 - 109)	0.000
**FEV1**	374	2.1 (1.6 - 2.7)		2.6 (2.3 - 3.0)		1.97 (1.4 - 2.5)	0.000
**FEV1%**	374	88 (65 - 105)		103 (91.5 - 115.5)		79 (60 - 100)	0.000
**FEV1/FVC ratio**	374	0.76 (0.66 - 0.84)		0.82 (0.74 - 0.88)		0.73 (0.63 - 0.83)	0.000
**Lung function**	374						
**Normal**		201 (53.7)		76 (86.4)		125 (43.7)	0.000
**Poor**		173 (46.3)		12 (13.6)		161 (56.3)	
**Post-measurement**			9		117		
**FVC**	126	2.6 (2.2 - 3.4)		3.1 (2.8 - 3.6)		2.62 (2.2 - 3.4)	0.050
**FVC %**	126	99.5 (80 - 112)		117 (111 - 120)		97 (80 - 111)	0.002
**FEV1**	126	1.8 (1.3 - 2.3)		2.3 (2.1 - 2.5)		1.73 (1.29 - 2.2)	0.010
**FEV1%**	126	73.5 (56 - 89)		98 (92 - 103)		71 (56 - 87)	0.000
**FEV1/FVC ratio**	126	0.65 (0.56 - 0.71)		0.74 (0.7 - 0.76)		0.64 (0.56 - 0.71)	0.006
**Methacholine challenge test**	361		85		277		
**Positive**		23 (6.4)		2 (2.4)		21 (7.6)	0.190
**Negative**		338 (93.4)		83 (97.6)		255 (92.1)	
**Skin prick, at least one allergen positive**	331		71		260	(0)	
**Yes**		310 (93.7)		67 (94.4)		243 (93.5)	1.000
**No**		21 (6.3)		4 (5.6)		17 (6.5)	
**Eosinophilic count**	374						
**AEC<500**		310 (82.9)		72 (81.8)		238 (83.2)	0.761
**AEC>=500**		64 (17.1)		16 (18.2)		48 (16.8)	
**Eosinophil count**	374	210 (110 - 390)		200 (110 - 410)		215 (100 - 390)	0.984
**Total BHQ score**	374	72 (49 - 98)	88	61.5 (32.5 - 78.5)	286	77.5 (53 - 102)	0.000
**Patient uses ICS**	370	34 (9.2)	87	3 (3.4)	283	31 (11)	0.034
**History of smoking**	374		88		286		
**Current smoker**		1 (0.3)		0 (0)		1 (0.3)	0.569
**Former smoker**		22 (5.9)		7 (8)		15 (5.2)	
**Never**		351 (93.9)		81 (92)		270 (94.4)	
**Secondary smoker**	374	20 (5.3)	88	4 (4.5)	286	16 (5.6)	1.000
**Relatives who have asthma**	372		87		285		
**Yes**		222 (59.7)		50 (57.5)		172 (60.4)	0.149

**No**		89 (23.9)		27 (31)		62 (21.8)	
**Don't know**		61 (16.4)		10 (11.5)		51 (17.9)	
**Exposed to firewood/ charcoal smoke**	374	344 (92)	88	76 (86.4)	286	268 (93.7)	0.027
**Use of kerosene for lighting or cooking**	374	374 (100)	88	12 (13.6)	286	37 (12.9)	0.865
**Depression status according to total PHQ-9 score**	374		88		286		
**No depression**		15 (4)		9 (10.2)		6 (2.1)	0.000
**Minimal depression**		96 (25.7)		27 (30.7)		69 (24.1)	
**Mild depression**		113 (30.2)		32 (36.4)		81 (28.3)	
**Moderate depression**		92 (24.6)		11 (12.5)		81 (28.3)	
**Moderately severe depression**		47 (12.6)		9 (10.2)		38 (13.3)	
**Severe depression**		11 (2.9)		0 (0)		11 (3.8)	
**Patient uses inhaler medication**	370	108 (29.2)	87	13 (14.9)	283	95 (33.6)	0.001
**Asthma triggers**	374		88		286		
**Upper respiratory infection**		371 (99.2)		87 (98.9)		284 (99.3)	0.514
**Exposure to household pets such as cats, dogs or poultry**		13 (3.5)		4 (4.5)		9 (3.1)	0.514
**Smoking or exposure to tobacco smoke**		356 (95.2)		81 (92)		275 (96.2)	0.115
**Strong emotions such as anger, excitement, anxiety**		223 (59.6)		45 (51.1)		178 (62.2)	0.063
**Cold weather**		341 (91.2)		82 (93.2)		259 (90.6)	0.448
**Drugs such those treating high blood pressure and pain killers like aspirin**		1 (0.3)				1 (0.3)	1.000
**Exercise**		116 (31)		21 (23.9)		95 (33.2)	0.097
**Dust**		359 (96)		83 (94.3)		276 (96.5)	0.361
**Strong smells or perfumes**		315 (84.2)		74 (84.1)		241 (84.3)	0.969
**Exposure at work**		13 (3.5)		2 (2.3)		11 (3.8)	0.741
**Other**		5 (1.3)		2 (2.3)		3 (1)	0.336
**None of the above**		0 (0)		0 (0)		0 (0)	NA
